# Three-Dimensional versus Two-Dimensional Evaluations of Cranial Asymmetry in Deformational Plagiocephaly Using a Three-Dimensional Scanner

**DOI:** 10.3390/children9060788

**Published:** 2022-05-27

**Authors:** Risa Kato, Nobuhiko Nagano, Shin Hashimoto, Katsuya Saito, Hiroshi Miyabayashi, Takanori Noto, Ichiro Morioka

**Affiliations:** 1 Department of Pediatrics and Child Health, Nihon University School of Medicine, Oyaguchi-kamimachi, Itabashi-ku, Tokyo 173-8610, Japan; kato.risa@nihon-u.ac.jp (R.K.); nagano.nobuhiko@nihon-u.ac.jp (N.N.); noto.takanori@nihon-u.ac.jp (T.N.); 2 Department of Pediatrics, Kasukabe Medical Center, Saitama 344-8588, Japan; fight.together.0119@gmail.com (S.H.); katsuya-saito@nifty.com (K.S.); miyabayashi@dr.memail.jp (H.M.); 3 Noto Children’s Clinic, Hikawadai, Nerima-ku, Tokyo 179-0084, Japan

**Keywords:** anterior symmetry ratio, cranial asymmetry, head deformity, plagiocephaly, posterior symmetry ratio

## Abstract

This study aimed to assess the measurement precision of a three-dimensional (3D) scanner that detects the geometric shape as surface data and to investigate the differences between two-dimensional (2D) and 3D evaluations in infants with deformational plagiocephaly. Using the 3D scanner that can perform both 2D and 3D evaluations, we calculated cranial asymmetry (CA) for the 2D evaluation, and the anterior symmetry ratio (ASR) and posterior symmetry ratio (PSR) for the 3D evaluation. Intra- and inter-examiner precision analyses revealed that the coefficients of the variation measurements were extremely low (<1%) for all variables, except CA (5%). In 530 infants, the coincidence rate of CA severity by the 2D evaluation and the 3D evaluation was 83.4%. A disagreement on severity was found between 2D and 3D evaluations in 88 infants (16.6%): 68 infants (12.8%) were assessed as severe by 2D evaluation and mild by the 3D evaluation, while 20 infants (3.8%) were evaluated as mild by 2D and severe by 3D evaluation. Overall, the 2D evaluation identified more infants as severe than the 3D evaluation. The 3D evaluation proved more precise than the 2D evaluation. We found that approximately one in six infants differed in severity between 2D and 3D evaluations.

## 1. Introduction

Head volume is obtained by computed tomography and magnetic resonance imaging, whereas the three-dimensional (3D) scanner detects the geometric shape of the object as surface data and calculates the ratio rather than the volume. There is currently no gold-standard measurement method for cranial asymmetry worldwide. A 3D evaluation method using a 3D scanning analyzer (3D scanner) was recently used to assess head deformity severity in infants because it is simple, safe, and convenient, and does not require the use of computed tomography or magnetic resonance imaging. 

Two-dimensional (2D) evaluation is a method for assessing head deformities using diagonal length differences in a single plane [1]. The cross-section with the maximum head circumference is used for the measurement plane [2,3], such as the maximum posterior curved plane of the occiput [4], the plane passing through the contralateral lambda suture from the frontal junction point [5], and the plane at the inferior cranial level (superior orbital rim level) [6]. Some reports indicated that 2D evaluations have low measurement variability, but high interobserver variability [7,8]. Another report highlighted a potential error in 2D plane selection [9]. In addition, the values calculated for different 2D cross-sections of the same infant differed [10]. Therefore, it is possible that 2D evaluations alone do not sufficiently assess the entire 3D cranial structure. In particular, the occiput, the main positional deformational plagiocephaly site, may be difficult to assess using 2D evaluations. 

The 3D evaluation, which has been used previously [4,11], is expected to be able to evaluate cranial shape in a more detailed and multifaceted way. However, its measurement precision has not been yet analyzed on 3D images acquired with the 3D scanner device used in this present study. This study was the first to assess the measurement precision of the 2D and 3D evaluations using the 3D scanner that can perform both kinds of evaluations (Study 1). 

In our previous study of infants (aged 4–8 months) with severe deformational plagiocephaly, 66% did not improve without cranial helmet therapy [12]. Therefore, treatment decisions at the appropriate time are important for infants with severe plagiocephaly. To enable more effective therapeutic interventions, it is necessary to clarify the correlations and differences between 2D and 3D evaluation methods. The 2D evaluation is currently popular for the classification of severe deformational plagiocephaly. Therefore, the second study aim was to investigate the differences between 2D and 3D evaluations using the 3D scanner and to clarify the diagnostic value of 2D evaluations (Study 2).

## 2. Materials and Methods

### 2.1. Study Design and Subjects

Two studies were conducted. In Study 1, to measure precision, we assessed the repeatability (intra- and inter-examiner precision analyses) of the Artec Eva 3D scanner that can perform the 2D and 3D evaluations (Artec, Inc., Luxembourg, Luxembourg). In Study 2, we examined the differences between 2D and 3D evaluations using the 3D scanner, and clarified the diagnostic value of the 2D evaluations.

Study 1: The randomly selected participant was a Japanese adult. To determine the repeatability of the values obtained using the 3D scanner, intra- and inter-examiner precision analyses were performed. The following variables were examined: cranial length, cranial width, head circumference, and cranial asymmetry (CA) in 2D evaluations, and anterior symmetry ratio (ASR) and posterior symmetry ratio (PSR) in 3D evaluations.

Study 2: This study included infants who visited three hospitals (Nihon University Itabashi Hospital, Kasukabe Medical Center, and Noto Children’s Clinic) for medical checkups or head deformities between April 2020 and April 2021, and their cranial shapes were measured using a 3D scanner. The following variables were examined: CA in 2D evaluations and ASR and PSR in 3D evaluations.

A 360° scan of the cranial shape, including both ears, was performed using a 3D scanner. However, this has not yet been approved as a medical device by the Pharmaceuticals and Medical Devices Agency in Japan. Written informed consent was obtained from the parents or guardians of all participants. This study was approved by the ethics committees of the participating institutions (Kasukabe Medical Center and Noto Children’s Clinic: approval number 2019-032; 12 March 2020; and Nihon University Itabashi Hospital: approval number RK-200512-2; 22 May 2020). 

### 2.2. Data Acquisition Using the 3D Scanner

Head deformities were imaged using an Artec Eva 3D scanner. Before the scanning, the infant’s head was protected using a stocking cap to avoid hair disturbance, and all hair was set inside the cap. With the caregivers holding the infants, 360-degree scans were performed. From a distance of 40 cm to 1 m from the infant’s head, the 3D scanner continuously and intermittently shone lights at a maximum of 16 times per second. The scanner detected light deflected from the surface of the head and recorded information on unevenness and color. The frame rate per second constantly changed, depending on the computer environment during shining. The measurement time, including preparation, was approximately 5 min.

### 2.3. Data Analysis Method

The obtained data were analyzed using Artec Studio image analysis software (Artec, Inc., Luxembourg, Luxembourg) to obtain 3D images and determine cranial shape. The entire 3D dataset was constructed by combining the overlapping regions in successive scanned frames. We measured the following variables from the 3D scanned images: CA using the 2D evaluation method and ASR and PSR using the 3D evaluation method.

#### 2.3.1. How to Calculate ASR and PSR

**Figure 1:**
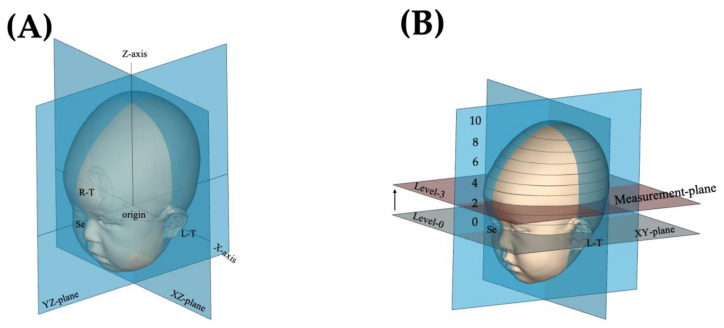
Three-dimensional images. (**A**) The methods by which the X-axis, Y-axis, and Z-axis were determined. (**B**) The base XY plane runs through the SE and the left and right TRs. Ten equidistant and parallel cross-sections are conducted to the cranium superior (level 10) from the base XY plane (level 0). The figures were cited from the figures by Noto et al. [12]. SE, at the most concave point in the soft tissue at the nasofrontal angle between the forehead slope and the proximal nasal bridge, TR, at the upper margin of the tragus.

Figure 1 shows a 3D quantified global view. First, the sellion (SE: at the most concave point in the soft tissue at the nasofrontal angle between the forehead slope and proximal nasal bridge) [13,14,15] and the left and right tragions (TRs: at the upper margin of the tragus) were determined. Next, we determined the basic cross-section (XY plane) as the plane that passed through the SE and the left and right TRs. The midpoint of both TRs was defined as the origin. After setting these landmarks and the basic plane, the line passing through the SE and the origin was defined as the Y-axis. The X-axis was defined as the line perpendicular to the Y-axis that crosses the origin on the basic plane. The Z-axis was defined as the line perpendicular to the XY plane that crosses the origin [11,12,16]. From the XY plane (level 0), 10 equidistant, parallel cross-sections through the upper part of the skull (level 10) were constructed, and cross-sections from levels 2 to 8 were used to calculate the volume of the entire cranium, excluding the soft tissues of the ear and face [17] (Figure 1B).

**Figure 2:**
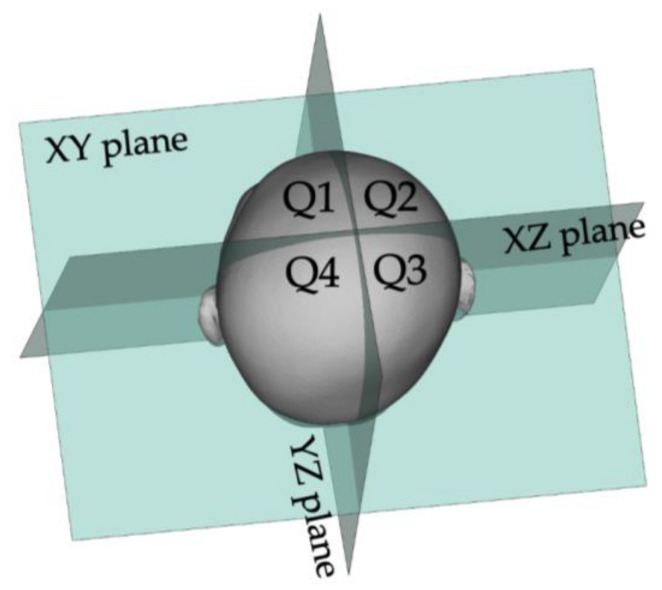
Four quadrant volumes. The total volume was divided into four quadrants along the XZ and YZ planes. Each quadrant volume was used to quantitatively define the bilateral symmetry ratio of ASR (Q1 volume/Q2 volume, or vice versa × 100, %) and PSR (Q3 volume/Q4 volume, or vice versa × 100, %); a value where either the Q1 volume/Q2 volume, Q3 volume/Q4 volume, or vice versa, is <100% was chosen. ASR, anterior symmetry ratio; PSR, posterior symmetry ratio.

The total volume was divided into four quadrants using planes passing through the X- and Y-axes and containing the Z-axis (XZ and YZ planes) (Q1, anterior left; Q2, anterior right; Q3, posterior right; Q4, posterior left). Finally, each quadrant volume was used to quantitatively define the bilateral symmetry ratio of ASR (Q1 volume/Q2 volume, or vice versa × 100 %) and PSR (Q3 volume/Q4 volume, or vice versa × 100%), a value where either the Q1 volume/Q2 volume, Q3 volume/Q4 volume, or vice versa, was <100% was chosen [11,16,18] (Figure 2).

#### 2.3.2. Cross-Sectional Level for 2D Measurement 

The largest head circumference should ideally be used as the basic 2D measurement plane [3,4,16]. In this study, however, to unify the level of the measurement plane, cross-Section 3 (Level 3) was selected as the standard measurement plane of the 2D evaluation [10,12,17] (see Figure 1B).

#### 2.3.3. How to Calculate CA

**Figure 3:**
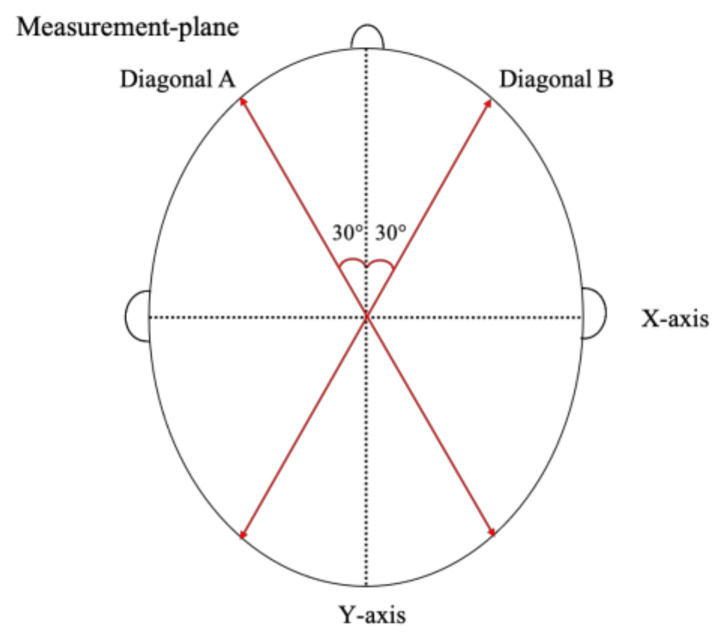
Measuring cranial asymmetry (CA). Two diagonals (A and B) are drawn 30° from the Y-axis on level 3. CA (mm) = Diagonal A–Diagonal B. This figure was cited from reference [12].

According to Loveday et al. [19], two diagonals drawn 30° from the Y-axis were measured. The CA was calculated using these diagonals. The CA was calculated as diagonal A−diagonal B (mm) (Figure 3).

### 2.4. Severity Classifications

The severity of the 3D evaluation was defined as follows: mild if ASR ≥ 80.5%, or severe if ASR < 80.5%; mild if PSR ≥ 80.5%, or severe if PSR < 80.5%; and mild if both ASR and PSR were ≥80.5 %, or severe if ASR or PSR was <80.5% [11] (Supplementary Table S1). The severity of the CA-based 2D evaluation was defined as mild (CA = 0–12 mm), or severe (CA > 12 mm) [7,12,20,21].

### 2.5. Study Methods and Statistical Analyses

Study 1:

(1) Age, height, weight, and head circumference at the measurement date were collected.
(2) To determine the repeatability of the 3D scanner, six replicates of the scans were performed by one examiner (intra-examiner precision analysis). Six replicate scans were then performed by six different examiners (one scan per examiner; inter-examiner precision analysis). Mean, standard deviation (SD), and coefficient of variation (CV) were then calculated.

Study 2:

(1) Perinatal and neonatal factors included sex, gestational weeks at birth, birth weight, age at the time of measurement, mode of delivery, and intrauterine position.
(2) The values of head circumferences measured by the 3D scanner and by trained nurses were compared using bivariable normal ellipses, and regression analysis (correlation coefficient [r] was calculated).
(3) Distribution maps of the ASRs, PSRs, and CAs are shown. Each infant was classified by severity (mild or severe) using CA for the 2D evaluation, and ASR alone, PSR alone, or ASR and PSR for the 3D evaluation. The coincidence rate was analyzed, and the indicative variable in the 3D evaluation that showed the maximum coincidence rate was determined.
(4) Finally, infants whose severity assessed differently, especially the group identified as mild on the 2D evaluation and severe on the 3D evaluation, were selected. Using CA on planes other than level 3 at levels 2–8, we investigated whether there was a change in the coincidence rate.

Statistical calculations were performed using JMP (ver 14.0; SAS Institute Inc., Cary, NC, USA), when needed.

## 3. Results

### 3.1. Study 1

#### 3.1.1. Subject

The randomly selected subject was a 28-year-old Japanese male with a height of 180.2 cm, weight of 74 kg, and head circumference of 582 mm.

#### 3.1.2. Measurement Precision

The mean ± SD and CV for the intra- and inter-examiner precision analyses are shown in Table 1. The CV values were extremely low for all measurement variables, except CA.

### 3.2. Study 2

#### 3.2.1. Clinical Characteristics

This study included 530 infants (*n* = 257 at Nihon University Itabashi Hospital, *n* = 69 at Kasukabe Medical Center, and *n* = 204 at Noto Children’s Clinic). The median age at the time of measurement was three months. Detailed clinical characteristics of the infants are shown in Table 2.

#### 3.2.2. Correlation of Measurement Values

**Figure 4:**
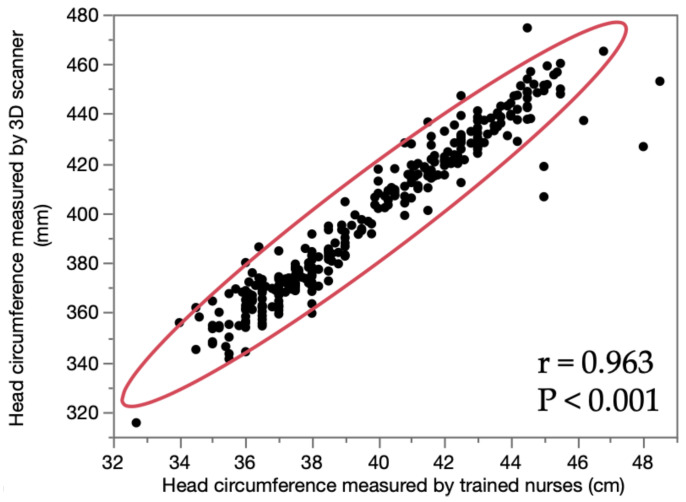
Correlation of the measurement values. Correlation of head circumference values measured by trained nurses versus the 3D scanner.

To determine the correlation between the head circumference values measured by trained nurses and the 3D scanner, the measurements of 321 infants were compared. A strong correlation was found between the two values (r = 0.963, *p* < 0.001; Figure 4). 

#### 3.2.3. Severity Classifications

**Figure 5:**
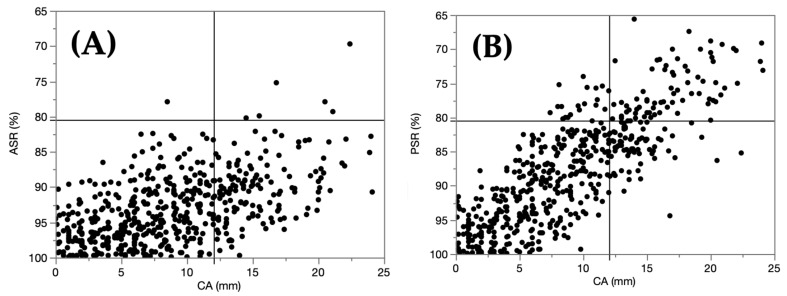
The distribution map of ASRs and CAs **(A)** and PSRs and CAs **(B).** ASR, anterior symmetry ratio; CA, cranial asymmetry; PSR, posterior symmetry ratio.

The severity coincidence rates between the 2D evaluation by CA and the 3D evaluation by ASR or PSR were evaluated using the different selected thresholds, such as 75.0%, 80.5%, 82.5%, and 85.0%. The severity coincidence rates were 77.9%, 83.4%, 83.8%, and 80.8%, respectively (Supplementary Table S1), indicating that the thresholds of 80.5% and 82.5% had approximately the same coincidence rate. Therefore, the threshold of 80.5% was selected for Study 2. Figure 5 shows the distribution maps of ASRs, PSRs, and CAs.

Table 3 shows the severity classifications using the 2D evaluation by CA and 3D evaluation by ASR alone, PSR alone, and ASR/PSR. A total of 389 infants (73.4%) had the same severity classifications for CA and ASR in the 3D evaluation (Table 3A). A total of 438 infants (82.6%) had the same severity of CA and PSR in the 3D evaluation (Table 3B). As shown in Table 3C, the severity by CA in the 2D evaluation and that by ASR or PSR in the 3D evaluation agreed with each other for 442 infants (83.4%). More infants were determined as severe using the 2D versus the 3D evaluation (27.5% and 18.5%, respectively).

As shown in Table 3C, there were 68 infants (12.8%) in the group with severe 2D and mild 3D evaluation findings and 20 infants (3.8%) in the group with mild 2D and severe 3D evaluation findings, showing more severe results for the 2D evaluation by CA.

#### 3.2.4. CAs of Other Levels

Twenty infants, who showed more severe results in the 3D evaluation than the 2D evaluation, were selected for the further study. We investigated the CA on the planes at levels 2–8, other than level 3, in 20 infants. Eight (40%) of the 20 infants had severe classifications when other levels were evaluated (the yellow highlights show CA > 12 mm in Table 4), indicating consistency with the severity in the 3D evaluation.

## 4. Discussion

This study had several novel findings. First, it demonstrated the intra- and inter-examiner precision of the Artec Eva 3D scanner. Second, the severity assessments differed slightly between the 2D and 3D evaluations of head deformity; 16.6% were assessed differently (88/530 infants). In particular, 3.8% were evaluated as mild on the 2D evaluation and severe on the 3D evaluation (20/530 infants). Third, the coincidence rate of the severity increased at a higher level (levels 4–7) from the standard measurement plane (level 3), showing that the level that usually reflects the largest head circumference does not necessarily represent the maximum deformity.

The measurement precision using intra- and inter-examiner precision analyses with the Artec Eva 3D scanner showed extremely high repeatability. Compared with the other variables, the CV of CA was high. This may have been due to hair disturbances. To avoid this effect, the hair was placed inside the cap as much as possible. In addition, a significant strong and positive correlation was noted between the measurements of head circumference by trained nurses and the 3D scanner.

A 2D evaluation is used to classify severe deformational plagiocephaly. Our study found that 83.4% of infants had the same severity on the 2D and 3D evaluations of head deformities. In the 3D evaluation, if PSR was used, more infants were identified as severe than with the 2D evaluation. Many positional head deformities are caused by sleep positioning [22]. Our results are consistent with those of a previous report by Argenta et al. in that the occipital region usually shows CA [23]. Although 16.6% of the infants were assessed differently in the current study, 2D evaluations can generally substitute for 3D evaluations.

Of the 88 infants assessed differently, 20 infants (23%) were evaluated as mild by 2D and severe by 3D evaluation. Thus, the infants subjected to the 2D evaluation may miss the appropriate timing for cranial helmet therapy. Therefore, we focused on the group with mild 2D and severe 3D evaluation results (*n* = 20) and performed further studies. We found that when the 2D evaluation was used at a higher level (levels 4–7), the severity consistency increased (40%). A previous report suggested that very severe occipital asymmetry could occur in the absence of frontal asymmetry associated with occipital plagiocephaly [24]. In this case, the CA value should be low because the CA value includes the frontal and occipital regions of the cranium. Therefore, in some infants, even when 2D evaluation classifies the condition as mild, a head deformity may be present at higher levels from the measurement level. Because the 3D evaluation can evaluate the asymmetry of the head shape from multiple aspects, if possible, it would be suitable to use both 2D and 3D evaluations to evaluate the exact severity. We believe that referring to the CA values at levels other than the maximal cranial circumference can lead to a multifaceted evaluation of cranial asymmetry. If it is difficult to measure on multiple 2D measurement planes, other parameters of 2D evaluation, such as the cranial vault asymmetry index and oblique cranial length ratio, may be useful [20,24].

To conduct the current study, we searched the literature for a definition of severity used in 3D evaluations, but those we found were inconsistent [7,11,12,20,21]. Although there was no firm professional consensus for the severity threshold in 3D evaluations, a certain criterion was needed to analyze the difference between the 2D and 3D evaluation methods in the current study. We analyzed the severity coincidence rates between 2D evaluation by CA, and 3D evaluation by ASR or PSR using the different selected thresholds, such as 75.0%, 80.5%, 82.5%, and 85.0%. The thresholds of 80.5% and 82.5% showed approximately the same coincidence rates (Supplementary Table S1).

Many institutions in Japan face hurdles in using 3D techniques for diagnostics because 3D scanners are expensive. Compared to a scanner that can take measurements within a few milliseconds from several spatial directions and create a stereophotogrammetric dataset, the Artec Eva 3D scanner used in the current study has the advantage of being mobile, although it takes a little longer to take images, and artifacts occur due to infant movements [25]. As shown in our measurement precision analyses, the 3D scanner used in our current study was useful for considering human errors between measurers in 2D evaluations [7,8]. Normal reference values of the measurement variables obtained by the 3D scanner in 1-month-old healthy Japanese male or female infants were shown in our recently published report [26].

Our study has some limitations. First, because 2D and 3D evaluations have different aspects, it was not possible to directly examine a correlation. Second, in the current study, we compared the CA values as representative 2D and 3D evaluation variables. Future studies are needed to evaluate the relationship between the cranial vault asymmetry index, which can appropriately express 3D shapes, and 3D evaluation results.

## 5. Conclusions

The Artec Eva 3D scanner provides high measurement precision. Using this scanner, this study found that the severity of five of six infants was the same between the 2D and 3D evaluations of head deformity, indicating that the former can generally be used as a substitute for the latter. Meanwhile, one of six infants were assessed differently. Therefore, if possible, it would be ideal to use both methods to evaluate head deformity severity.

## Figures and Tables

**Table 1:** Intra- and inter-examiner precision analyses of the 3D scanner results.

	Intra-Examiner		Inter-Examiner	
	Mean ± SD	CV (%)	Mean ± SD	CV (%)
	*n* = 6		*n* = 6	
Cranial length, mm	202.3 ± 0.5	0.26	203.0 ± 0.8	0.39
Cranial width, mm	171.7 ± 0.2	0.13	171.8 ± 0.3	0.16
Head circumference, mm	582.9 ± 1.2	0.20	582.9 ± 0.7	0.12
CA, mm	10.0 ± 0.6	5.62	10.0 ± 0.5	5.33
ASR, %	98.7 ± 0.2	0.24	98.6 ± 0.2	0.15
PSR, %	83.4 ± 0.4	0.50	83.7 ± 0.3	0.38

ASR, anterior symmetry ratio; CA, cranial asymmetry; CV, coefficient of variation; PSR, posterior symmetry ratio; SD, standard deviation.

**Table 2:** Infants’ clinical characteristics (*n* = 530).

Male, %	315 (59.4)
Gestational age at birth, weeks	39 (37–42)
Birth weight, grams	3024 (1639–4144)
Age at measurement, months	3 (0–47)
Mode of delivery	
Vaginal	313 (59.0)
Caesarean	161 (30.3)
Vacuum	45 (8.4)
Forceps	11 (2.0)
Intrauterine position	
Cephalic	494 (93.2)
Breech	31 (5.8)
Transverse	5 (0.9)

Data for gestational age at birth, birth weight, and age at measurement are shown as median (min–max). Others are shown as number (percentage).

**Table 3:** Severity classification using 2D evaluation by CA and 3D evaluation by ASR or PSR.

(A)		CA		
		Mild	Severe	Total
ASR ≥ 80.5%	Mild	383 (72.3%)	140 (26.4%)	523 (98.7%)
ASR < 80.5%	Severe	1 (0.2%)	6 (1.1%)	7 (1.3%)
	Total	384 (72.5 %)	146 (27.5 %)	530
The severity coincidence rate was 73.4% (389/530).				
(**B**)		**Mild**	**Severe**	**Total**
PSR ≥ 80.5%	Mild	365 (68.9%)	73 (13.8%)	438 (82.6%)
PSR < 80.5%	Severe	19 (3.6%)	73 (13.8%)	92 (17.4%)
	Total	384 (72.5%)	146 (27.5%)	530
The severity coincidence rate was 82.6% (438/530).				
(**C**)		**Mild**	**Severe**	**Total**
ASR and PSR ≥ 80.5%	Mild	364 (68.7%)	68 (12.8%)	432 (81.5%)
ASR or PSR < 80.5%	Severe	20 (3.8%)	78 (14.7%)	98 (18.5%)
	Total	384 (72.5%)	146 (27.5%)	530
The severity coincidence rate was 83.4% (442/530).				

ASR, anterior symmetry ratio; CA, cranial asymmetry; PSR, posterior symmetry ratio.

**Table 4:** CA values at levels 2–8 in the group with mild 2D and severe 3D evaluation findings (*n* = 20 in Table 3C).

No.				CA (mm)			
	Level 2	Level 3 (Standard)	Level 4	Level 5	Level 6	Level 7	Level 8
1	8	9.0	8.3	8.9	9.4	8.2	5.5
2	10.9	11.5	13.9	15.9	15.7	13.7	10.9
3	9.4	9.6	9.8	10.9	10.7	9.4	7.7
4	7	8.8	8.2	8.6	9.4	10.0	8.6
5	8.3	8.4	9.4	11.5	11.8	10.3	7.4
6	8.9	10.1	11.5	12.5	12.2	11.3	8.8
7	9.7	11.1	11.1	11.2	10.9	9.5	7.9
8	10.7	11.7	12.3	12.6	11.8	10.1	8.4
9	12.2	10.3	14.2	15.7	15.6	13.4	8.8
10	6.2	10.0	12.2	14.7	13.6	11.7	9.1
11	5.5	7.4	6.3	8.0	9.4	9.1	7.4
12	7.3	8.3	9.1	9.4	9.6	7.8	6.4
13	8.5	8.5	10.1	9.6	8.6	7.2	5.6
14	7.1	9.7	7.8	10.3	12.2	11.9	10.7
15	8.8	12.0	16.9	18.7	17.7	15.3	11.7
16	10.2	11.0	11.1	11.3	11.1	10.3	8.5
17	8.5	11.0	10.7	12.4	12.1	10.5	9
18	6.5	8.0	9.8	10.8	10.8	10.7	9.8
19	6.6	8.6	8.0	9.4	10.0	9.6	8.5
20	7.8	8.1	9.6	10.6	10.8	9.5	8.3

Yellow highlights represent severe classifications by 2D evaluation (CA > 12 mm). 2D, two-dimensional; 3D, three-dimensional; CA, cranial asymmetry.

## Data Availability

The data presented in this study are available upon request from the corresponding author.

## Supplementary Materials

The following supporting information can be downloaded at https://www.mdpi.com/article/10.3390/children9060788/s1: Table S1. Severity classification of two-dimensional evaluation by cranial asymmetry versus three-dimensional evaluation by anterior symmetry ratio or posterior symmetry ratio using different selected thresholds.
