# Supplementary material for: Three-Dimensional versus Two-Dimensional Evaluations of Cranial Asymmetry in Deformational Plagiocephaly Using a Three-Dimensional Scanner
Source: Children (Basel). 2022 May 27;9(6):788. doi: 10.3390/children9060788 (PMC9221621; doi:10.3390/children9060788)
Supplement: Supplementary file 1 [file children-09-00788-s001.zip › children-1735938-supplementary.pdf]

Supplementary Materials

Supplementary Table S1. Severity classification of two-dimensional evaluation by cranial asymmetry versus three-dimensional evaluation by anterior symmetry ratio or posterior symmetry ratio using different selected thresholds.

(A) 75.0%		CA		
		Mild	Severe	Total
ASR and PSR ≥ 75.0%	Mild	383 (72.2%)	116 (21.9%)	499 (94.2%)
ASR or PSR < 75.0%	Severe	1 (0.2%)	30 (5.7%)	31 (5.8%)
	Total	384 (72.5%)	146 (27.5%)	530

The coincidence rate of severity was 77.9% (413/530).

(B) 80.5%		CA		
		Mild	Severe	Total
ASR and PSR ≥ 80.5%	Mild	364 (68.7%)	68 (12.8%)	432 (81.5%)
ASR or PSR < 80.5%	Severe	20 (3.8%)	78 (14.7%)	98 (18.5%)
	Total	384 (72.5%)	146 (27.5%)	530

The coincidence rate of severity was 83.4% (442/530).

(C) 82.5%		CA		
		Mild	Severe	Total
ASR and PSR ≥ 82.5%	Mild	349 (65.8%)	51 (9.6%)	400 (75.5%)
ASR or PSR < 82.5%	Severe	35 (6.6%)	95 (17.9%)	130 (24.5%)
	Total	384 (72.5%)	146 (27.5%)	530

The coincidence rate of severity was 83.8% (444/530).

(D) 85.0%		CA		
		Mild	Severe	Total
ASR and PSR ≥ 85.0%	Mild	297 (56.0%)	15 (2.8%)	312 (58.9%)
ASR or PSR < 85.0%	Severe	87 (16.4%)	131 (24.7%)	218 (41.1%)
	Total	384 (72.5%)	146 (27.5%)	530

The coincidence rate of severity was 80.8% (428/530).

ASR, anterior symmetry ratio; CA, cranial asymmetry; PSR, posterior symmetry ratio
